# Timing Matters: Early Versus Delayed Rehabilitation After Total Knee Arthroplasty and Its Impact on Functional Recovery—A Systematic Review

**DOI:** 10.3390/jfmk11020233

**Published:** 2026-06-09

**Authors:** Félix Menéndez-Vega, Sandra Núñez-Rodríguez, Jerónimo Javier González-Bernal, Jessica Fernández-Solana, Pedro Aparicio de Águeda, Mirian Santamaría-Peláez

**Affiliations:** 1 Faculty of Health Sciences, University Isabel I, 09003 Burgos, Spain; felix.menendez@ui1.es (F.M.-V.); sandra.nunez@ui1.es (S.N.-R.); 2 Faculty of Health Science, University of Burgos, 09001 Burgos, Spain; jfsolana@ubu.es (J.F.-S.); mspelaez@ubu.es (M.S.-P.); 3 Rehabilitation Service, Hospital Universitario de Burgos, 09006 Burgos, Spain; paparicioa@saludcastillayleon.es

**Keywords:** total knee replacement, postoperative care, physiotherapy timing, functional outcomes, recovery trajectory, joint mobility

## Abstract

**Background:** Total knee arthroplasty (TKA) is widely used to treat advanced knee osteoarthritis, yet the optimal timing for initiating postoperative rehabilitation remains unclear, particularly regarding its impact on short- and long-term functional outcomes. **Objective:** This study aimed to systematically review and compare earlier versus later initiation of structured postoperative rehabilitation following primary TKA according to the timing definitions used in the available literature of structured postoperative rehabilitation after primary TKA and its effects on functional recovery. **Methods:** Electronic searches were performed in PubMed, Scopus, Web of Science, and ScienceDirect between January and February 2025. Studies were limited to human participants, published in English or Spanish from 2010 onwards. Eligible studies compared early versus delayed rehabilitation following primary TKA and reported at least one predefined outcome related to pain, patient-reported functional measures, range of motion, muscle strength, performance-based functional tests, or hospital length of stay. Study selection was performed independently by two reviewers, and methodological quality was assessed using the Joanna Briggs Institute critical appraisal tools. **Results:** A total of 662 records were identified, of which five studies (three randomized controlled trials, one prospective observational study, and one retrospective cohort study), including 185 participants, met the inclusion criteria. Early rehabilitation (typically initiated within the first postoperative hours to days) was associated with reductions in hospital length of stay ranging from approximately 1 to 2 days, lower early postoperative pain scores, greater short-term knee flexion gains, and improved early muscle strength compared with delayed rehabilitation protocols. However, no consistent differences were observed in medium- and long-term patient-reported functional outcomes across studies. No increase in postoperative complications was reported. **Conclusions:** Early initiation of rehabilitation after TKA appears safe and may enhance short-term recovery outcomes. However, no consistent long-term functional differences were observed between earlier and later rehabilitation initiation across the included studies. Further high-quality research with standardized definitions and long-term follow-up is required.

## 1. Introduction

Total knee arthroplasty is primarily indicated in patients with advanced knee osteoarthritis who experience severe pain, functional limitation, and reduced quality of life despite conservative treatment [1]. The decision to proceed with surgery is typically based on radiographic severity, symptom burden, and failure of non-surgical interventions [1].

Epidemiologically, TKA is most frequently performed in older adults, particularly women, with increasing incidence due to population aging and rising obesity rates [2]. Postoperatively, patients commonly present with pain, joint stiffness, muscle inhibition, and reduced mobility, which necessitate early rehabilitation interventions to restore function [3].

Postoperative rehabilitation typically includes early mobilization, range-of-motion exercises, muscle strengthening, gait training, and progressive functional activities. In the acute phase, emphasis is placed on pain control, joint mobility, and early ambulation, whereas later stages focus on strength recovery, functional independence, and return to daily activities [4,5].

Early postoperative rehabilitation, defined as the initiation of structured mobilization and physiotherapy within the first hours or days after surgery, constitutes a core component of contemporary recovery pathways following total knee arthroplasty (TKA) [6]. Total knee arthroplasty is among the most frequently performed orthopedic procedures worldwide for the treatment of end-stage knee osteoarthritis, and its incidence continues to increase due to demographic aging and rising obesity prevalence [7]. Although total knee arthroplasty is highly effective in relieving pain and improving functional status, postoperative recovery trajectories remain heterogeneous and are influenced by multiple perioperative determinants, including the timing of rehabilitation initiation [8].

The immediate postoperative phase after TKA is characterized by acute inflammation, joint effusion, nociceptive sensitization, and substantial quadriceps inhibition [9,10]. Arthrogenic muscle inhibition contributes to rapid strength loss, impaired voluntary activation, altered gait patterns, and delayed achievement of functional milestones [11,12]. Prolonged immobilization may exacerbate these impairments, increasing the risk of joint stiffness, delayed range of motion (ROM) recovery, thromboembolic events, and extended hospital stay [4,13]. From a mechanistic perspective, early mobilization may mitigate neuromuscular inhibition, enhance synovial fluid distribution, promote periarticular tissue remodeling, and accelerate restoration of weight-bearing capacity and functional independence [14].

Enhanced Recovery After Surgery (ERAS) protocols in orthopedic surgery advocate early ambulation, often within the first 24 h postoperatively, as a strategy to reduce complications and shorten length of stay [5,15]. However, the operational definition of “early” versus “delayed” rehabilitation varies considerably across studies. Some authors define early rehabilitation as mobilization on the day of surgery, whereas others consider initiation within 48–72 h as early [4,16]. Therefore, this review adopts a pragmatic definition of rehabilitation timing based on the operational definitions used in each included study, rather than applying a fixed universal cutoff. This heterogeneity in timing thresholds, combined with variability in intervention characteristics (frequency, supervision, intensity, therapeutic modalities), complicates inter-study comparisons and limits the formulation of standardized clinical recommendations [17].

Beyond biomechanical and functional outcomes, rehabilitation timing may influence postoperative pain trajectories and opioid consumption [6,15]. Effective pain control is essential to enable active participation in rehabilitation; however, opioid-based strategies are associated with adverse events, prolonged use, and potential dependency [18,19]. Additionally, earlier rehabilitation may impact secondary outcomes including hospital length of stay, readmission rates, complication incidence, healthcare utilization, and patient-reported outcome measures (PROMs) [15,20].

Despite widespread clinical endorsement of early mobilization principles, uncertainty persists regarding the magnitude, consistency, and durability of its benefits compared with delayed initiation. Some studies report superior early ROM recovery, faster functional milestone attainment, and shorter hospitalization, whereas others demonstrate minimal differences in mid- or long-term outcomes [16,21,22]. Differences in study design, patient selection criteria, perioperative analgesia protocols, discharge pathways, and outcome definitions further contribute to inconsistency in the literature [10,17].

Given the clinical and economic implications of optimizing postoperative recovery after TKA, a comprehensive and methodologically rigorous synthesis of the available evidence is warranted. Clarifying whether earlier initiation of rehabilitation confers clinically meaningful advantages over delayed protocols is essential to inform perioperative care pathways and guide evidence-based decision-making.

Therefore, the objective of this systematic review is to identify, critically appraise, and synthesize the available evidence comparing early versus delayed initiation of postoperative rehabilitation following total knee arthroplasty. Specifically, this review aims to evaluate the effects of rehabilitation timing on pain, opioid consumption, range of motion, functional recovery, length of hospital stay, complications, and patient-reported outcomes, in order to provide robust evidence to support clinical and organizational decision-making.

Based on current evidence, we hypothesized that earlier initiation of structured rehabilitation would improve short-term functional outcomes without necessarily influencing long-term recovery. The guiding question of this review was whether early rehabilitation initiation leads to superior functional outcomes compared with delayed initiation following TKA.

## 2. Materials and Methods

### 2.1. Study Design and Research Question

This systematic review was conducted in accordance with the Preferred Reporting Items for Systematic Reviews and Meta-Analyses (PRISMA) guidelines [23]. The review protocol was prospectively registered in The International Prospective Register of Systematic Reviews (PROSPERO; registration number: CRD420261358594). The review methodology was defined a priori and followed a structured protocol to identify, screen, and synthesize the available scientific evidence on the impact of rehabilitation timing after total knee arthroplasty on functional outcomes. Due to heterogeneity in study designs, rehabilitation protocols, outcome measures, and follow-up durations, a quantitative meta-analysis was not feasible.

The clinically answerable research question was formulated using the PICO framework, given the heterogeneity in timing definitions across studies, the concepts of “earlier” and “later” rehabilitation initiation were interpreted pragmatically according to each study’s operational criteria rather than using a predefined universal temporal threshold. (Population, Intervention, Comparison, Outcomes; Table 1), as recommended for evidence-based research [24].

Importantly, the concept of “functional recovery” in this review is operationalized as a multidimensional construct encompassing patient-reported outcomes, joint range of motion, muscle strength, performance-based functional measures, and healthcare-related indicators such as length of hospital stay. This approach reflects the multifactorial nature of postoperative recovery after TKA, although it introduces heterogeneity that must be considered when interpreting the findings.

**Table 1:** PICO Format.

Population	Adult Patients (≥18 Years) Undergoing Primary Total Knee Arthroplasty.
Intervention	he early postoperative period (typically within the first 24–72 h after surgery, depending on study definitions).
Comparison	Later initiation of rehabilitation within the postoperative period, including initiation at 24–48 h, 48–72 h, postoperative day 7, or postoperative day 14, depending on study definitions.
Outcomes	Functional outcomes, including: Patient-reported outcome measures, knee range of motion, muscle strength and performance-based functional tests
Research question	In adult patients undergoing primary total knee arthroplasty, does earlier initiation of structured postoperative rehabilitation during the early postoperative period, compared with later initiation within the same postoperative phase, result in superior functional outcomes, including patient-reported functional scores, knee range of motion, muscle strength, and performance-based functional tests?

### 2.2. Search Strategy

A systematic search of the scientific literature was conducted in the following electronic databases: PubMed (MEDLINE), Scopus, Web of Science (Core Collection), and ScienceDirect. The complete search strategies used for each database are presented in Table 2. This search was performed between 18 of January and 21 of February 2025.

Search strategies were developed based on the PICO framework and were adapted to the specific syntax, indexing systems, and technical constraints of each database. The Web of Science platform allowed for a broader combination of terms, whereas ScienceDirect required a simplified strategy due to limitations in the number of Boolean operators. The restriction to studies published from 2010 onwards was applied to ensure relevance to contemporary surgical techniques and modern rehabilitation protocols. Language and date restrictions were applied to ensure inclusion of studies reflecting contemporary surgical techniques and modern rehabilitation protocols, as well as to allow accurate interpretation of findings. These adaptations were implemented to ensure optimal balance between sensitivity and specificity while maintaining conceptual consistency across databases.

The final search strategies for each database are presented below:

**Table 2:** Search strategy used, adapted to each of the databases.

Database	Search Strategy
Pubmed 68	((“total knee arthroplasty”[tiab] OR “total knee replacement”[tiab]) AND (“physical therapy”[tiab] OR physiotherap*[tiab]) AND (early[tiab] OR delayed[tiab] OR “early initiation”[tiab] OR “delayed initiation”[tiab]) AND (postoperative[tiab] OR post-operative[tiab]) AND (WOMAC[tiab] OR KOOS[tiab] OR “range of motion”[tiab] OR “timed up and go”[tiab])) NOT (review[pt] OR systematic review[tiab] OR meta-analysis[pt])
Scopus 176	TITLE-ABS-KEY((“total knee arthroplasty” OR “total knee replacement”) AND (“physical therapy” OR physiotherap*) AND (“early initiation” OR “delayed initiation” OR early OR delayed) AND (postoperative OR post-operative) AND (WOMAC OR KOOS OR “range of motion” OR “timed up and go”)) AND (LIMIT-TO(DOCTYPE, “ar”)) AND NOT TITLE-ABS-KEY(review OR “systematic review” OR meta-analysis) AND TITLE-ABS-KEY(functional)
ScienceDirect 278	“total knee arthroplasty” AND “physical therapy” AND postoperative AND (early OR delayed) AND (“range of motion” OR WOMAC) NOT review
WOS 140	TS = ((“total knee arthroplasty” OR “total knee replacement”) AND (“physical therapy” OR physiotherap*) AND (“early initiation” OR “delayed initiation” OR early OR delayed) AND (postoperative OR post-operative) AND (WOMAC OR KOOS OR “range of motion” OR “timed up and go”)) NOT TS = (review OR “systematic review” OR meta-analysis)

* Truncation symbol used to retrieve all words sharing the same root (e.g., physiotherapy, physiotherapist, physiotherapeutic).

The search was limited to human studies, published in English or Spanish, from 2010 onwards, to ensure clinical relevance and contemporary rehabilitation practices.

### 2.3. Elegibility Criteria, Study Selection, and Data Extraction

Studies were included if they met the following criteria: (1) original experimental, quasi-experimental, or observational research designs (including randomized controlled trials, non-randomized trials, and cohort studies); (2) adult participants (≥18 years) undergoing primary total knee arthroplasty; (3) explicit comparison between early and delayed initiation of postoperative rehabilitation; and (4) assessment of at least one functional outcome, such as patient-reported outcome measures (e.g., WOMAC, KOOS), knee range of motion, muscle strength, or performance-based functional tests.

Studies were excluded if they were systematic reviews, meta-analyses, narrative reviews, case reports, conference abstracts, editorials, or letters to the editor; if they included revision knee arthroplasty or mixed surgical populations without separate analysis for primary procedures; if the timing of rehabilitation initiation was not clearly defined; or if they focused exclusively on pharmacological, surgical, or technological interventions without a rehabilitation component. Animal and in vitro studies were also excluded.

The study by Liebs et al. included both total knee arthroplasty and total hip arthroplasty populations; however, only the subgroup-specific TKA data were extracted and analyzed in the present review.

**Figure 1:**
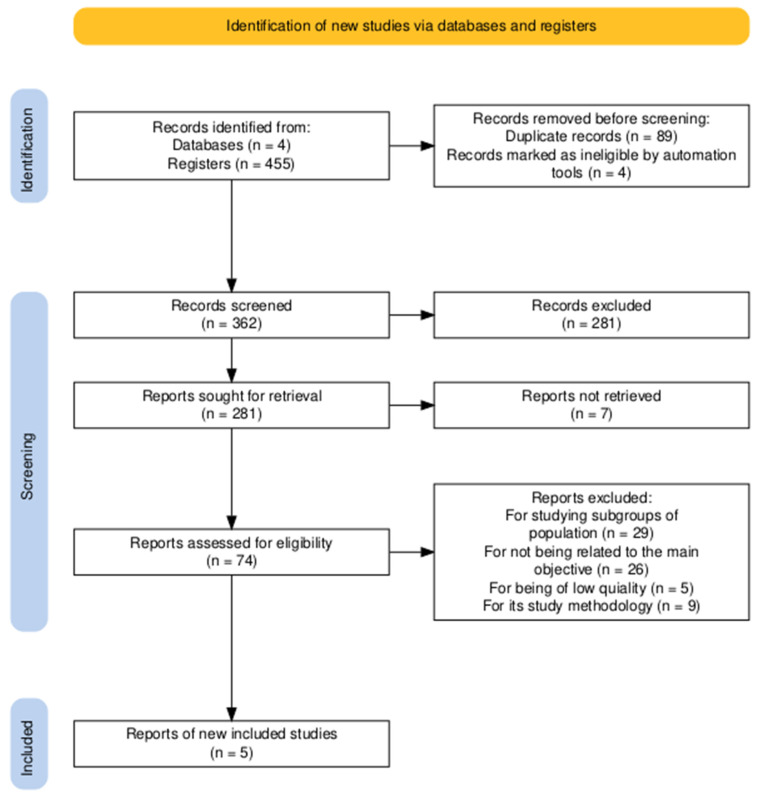
Flow-diagram for study selection.

The study selection process was conducted in two stages using Rayyan software [25]. First, titles and abstracts were independently screened by two reviewers to exclude clearly irrelevant records. Subsequently, full texts of potentially eligible studies were retrieved and assessed in detail against the predefined inclusion and exclusion criteria. Some records could not be retrieved due to access limitations or incomplete indexing, and were therefore excluded from the final selection process. Any discrepancies were resolved through discussion, and when necessary, a third reviewer was consulted. Disagreements during study selection were resolved through discussion between reviewers and, when necessary, consultation with a third reviewer. Consensus-based resolution was considered appropriate because all potentially eligible studies were independently reviewed and discussed before final inclusion. Figure 1 summarizes the results of the PRISMA-guided study selection process, including identification, screening, eligibility assessment, and final study inclusion.

Agreement between reviewers during study selection was assessed through discussion and consensus; however, no formal inter-rater reliability statistics were calculated.

Data extraction was performed using a standardized data collection form. The following information was extracted from each included study: authors and year of publication, country, study design, sample size and participant characteristics, definition of early and delayed rehabilitation, description of rehabilitation protocols, functional outcomes assessed and measurement instruments, and main findings.

### 2.4. Methodological Quality Assessment and Data Synthesis

Randomized controlled trials were assessed using the Joanna Briggs Institute critical appraisal checklist for randomized controlled trials, whereas the cohort study was assessed using the Joanna Briggs Institute checklist for cohort studies.

The methodological quality and risk of bias of the included studies were assessed using the Joanna Briggs Institute (JBI) critical appraisal tools, selecting the appropriate checklist according to the study design. The JBI critical appraisal tools were used to support structured methodological assessment rather than to exclude studies solely based on numerical scores. Prior to the formal assessment, a pilot evaluation was conducted between reviewers to ensure consistent application of the appraisal criteria. Each study was independently evaluated by two reviewers, and only studies achieving the predefined minimum quality threshold were included in the final synthesis.

Due to heterogeneity in study designs, rehabilitation protocols, outcome measures, and follow-up durations, a quantitative meta-analysis was not feasible. Therefore, the results were synthesized using a narrative approach, grouping studies according to rehabilitation timing and type of functional outcome assessed.

No formal prioritization of primary versus secondary outcomes was performed, which should be considered when interpreting the results.

## 3. Results

A total of 455 records were identified through database searching. After removal of duplicates, 362 records were screened by title and abstract. Subsequently, 81 full-text articles were assessed for eligibility. Of these, 74 were excluded for reasons including non-relevant population, absence of timing comparison, inappropriate study design, or insufficient outcome reporting. Finally, five studies met the inclusion criteria and were included in the qualitative synthesis.

Full-text articles excluded (*n* = 69):

- Population not relevant to the review question (*n* = 29);
- Not addressing rehabilitation timing as the primary objective (*n* = 26);
- Low methodological quality (*n* = 5);
- Inappropriate study design or methodology (*n* = 9).

Finally, a total of five studies met the eligibility criteria and were included in the final synthesis. These studies investigated the effects of early versus delayed initiation of postoperative rehabilitation following total knee arthroplasty. Table 3 also summarizes the main characteristics and key findings of the included studies, along with their methodological quality scores assessed using the Joanna Briggs Institute (JBI) critical appraisal tool.

Figure 1 presents the results of the PRISMA-guided study selection process, including identification, screening, eligibility assessment, and final study inclusion.

### 3.1. Study Characteristics

A total of five studies were included in this systematic review, comprising three randomized controlled trials [26,27,28,30], one retrospective cohort study [15] and one prospective observational study [28]. No included studies employed crossover or within-subject designs; all comparisons were conducted between independent patient groups receiving either early or delayed rehabilitation. The randomized trials primarily investigated the effects of early versus delayed initiation of structured postoperative rehabilitation following primary TKA, focusing on pain, range of motion, muscle strength, and functional recovery. The cohort study evaluated the impact of ultra-early physical therapy initiation on hospital length of stay and short-term functional outcomes.

The studies were conducted across different geographical regions, including Asia (Japan and Singapore), Europe (Germany and Spain), and South Asia (India). Sample sizes varied considerably, ranging from 100 participants in the smallest randomized trial [28] to 569 patients in the retrospective cohort study [15]. The multicenter randomized trial by Liebs et al. included 465 patients undergoing TKA or total hip arthroplasty (THA), although only the TKA subgroup was considered for the present review. Overall, the total number of participants across the included studies exceeded 1500 patients.

The mean age of participants ranged from approximately 62 to 75 years. Iwakiri et al. included the oldest population (mean age 75.3 ± 6.8 years), whereas Gadhavi et al. reported a younger cohort (mean age 62.4 ± 7.2 years) [15,26]. The remaining studies reported mean ages between 66 and 69 years. Women represented the majority of participants in most studies, reflecting the epidemiology of knee osteoarthritis, although the proportion of female participants varied between approximately 62% and 80%.

All included studies compared different timings of rehabilitation initiation within the early postoperative period. Definitions of “early” ranged from initiation within 12 h or 24 h after surgery [15,28,29] to postoperative day [26] and postoperative day 6 [27]. Comparator groups typically initiated rehabilitation between 48 and 72 h, postoperative day 7, or postoperative day 14. Despite variability in operational definitions, all studies explicitly evaluated the effect of earlier versus later rehabilitation onset.

Functional and clinical outcomes were assessed using validated instruments. Pain was measured using the Visual Analog Scale (VAS) in four studies. Knee range of motion was consistently evaluated using goniometric measurement. Muscle strength was assessed using manual muscle testing scales in the randomized trials by Labraca et al. and Gadhavi et al. [28,29]. Patient-reported functional outcomes were evaluated using standardized questionnaires, including the WOMAC [26,27,28], the Oxford Knee Score and Knee Society Score [15], and health-related quality of life measures such as the SF-36 [27]. Performance-based functional measures included the Tinetti test for gait and balance [29] and ambulatory distance at discharge [15]. Hospital length of stay was reported in four studies.

The interventions consisted of structured physiotherapy programs delivered in the inpatient setting, including early mobilization, range-of-motion exercises, muscle strengthening, gait training, and progressive functional activities. The intensity and structure of rehabilitation protocols were generally comparable between groups, with timing of initiation representing the principal difference.

Overall, the included studies demonstrated moderate methodological heterogeneity in terms of design, sample size, and follow-up duration. While some trials focused on short-term in-hospital outcomes at discharge, others included follow-up assessments extending to 3 months, 1 year, or 2 years postoperatively. Despite these differences, all studies shared the common objective of determining whether earlier initiation of structured postoperative rehabilitation after primary TKA leads to superior functional outcomes.

The methodological quality and risk of bias of the included studies are summarized in Table 4 and were assessed using the appropriate Joanna Briggs Institute (JBI) critical appraisal tool according to each study design. Randomized controlled trials were assessed using the JBI checklist for randomized controlled trials, whereas observational and cohort studies were assessed using the JBI checklist for cohort studies. The JBI critical appraisal tools were used to support structured methodological assessment rather than to exclude studies solely based on numerical scores. All randomized controlled trials achieved high scores (ranging from 8/11 to 10/11), indicating generally low to moderate risk of bias. The retrospective cohort study also met the quality criteria for its design, although it was inherently subject to potential selection bias and confounding.

**Table 4:** Methodological quality assessment of included studies using the Joanna Briggs Institute (JBI) critical appraisal tools.

Randomized Controlled Trials											
	Q1	Q2	Q3	Q4	Q5	Q6	Q7	Q8	Q9	Q10	Q11
[26]	+	+	+	−	−	+	+	+	+	+	+
[27]	+	+	+	−	−	+	+	+	+	+	+
[28]	+	+	+	−	−	−	+	+	+	+	+
[29]	+	+	+	+	−	+	+	+	+	+	+
**Cohort studies.**											
[15]	+	+	+	−	−	+	+	+	+	+	

Q1–Q11 correspond to the individual items of the Joanna Briggs Institute (JBI) critical appraisal checklists according to study design. For randomized controlled trials, the items evaluate randomization, allocation concealment, baseline comparability, blinding, follow-up completeness, outcome measurement, and statistical analysis. For cohort studies, the items assess group similarity, exposure measurement, confounding factors, follow-up adequacy, outcome assessment, and statistical analysis. Symbols: “+” = criterion met; “−” = criterion not met.

### 3.2. Description of the Characteristics of the Studies

The aim of this systematic review was to examine whether earlier initiation of structured postoperative rehabilitation after primary TKA, compared with later initiation, results in superior functional outcomes. The review focused on outcomes including patient-reported functional measures, knee range of motion, muscle strength, performance-based functional tests, and hospital length of stay.

A total of five studies were included in the synthesis, comprising four randomized controlled trials [26,27,28,29] and one retrospective cohort study [15], with an overall sample exceeding 1500 participants. Definitions of early rehabilitation varied across studies, ranging from initiation within 12 h or 24 h postoperatively to postoperative day 1 or day 6, while comparator groups initiated rehabilitation between 24–48 h, 48–72 h, postoperative day 7, or day 14. Follow-up duration ranged from discharge assessments to 24 months postoperatively. Due to heterogeneity in timing definitions, outcome measures, and follow-up periods, a formal meta-analysis was not performed; instead, a structured narrative synthesis was undertaken.

#### 3.2.1. Acute Postoperative Outcomes

Across the included studies, earlier initiation of structured postoperative rehabilitation after primary total knee arthroplasty (TKA) was consistently associated with improved short-term in-hospital outcomes.

Hospital length of stay was significantly reduced in patients who began rehabilitation earlier. Labraca et al. (2011) reported shorter hospitalization in patients initiating rehabilitation within 24 h compared with 48–72 h [29]. Similar findings were observed by Gadhavi et al. (2025) [28], Gadhavi et al. reported a 1.9-point reduction in VAS pain scores on postoperative day 3 in the early rehabilitation group compared with delayed rehabilitation (3.2 vs. 5.1; *p* < 0.001). Additionally, range of motion at week 2 was 13° greater in the early rehabilitation group (85° vs. 72°; *p* < 0.01), and hospital length of stay was reduced by 1.5 days (4.8 vs. 6.3 days; *p* < 0.001) [28]. In the retrospective cohort study by Thwin et al. (2024), initiation within 24 h was associated with a modest but statistically significant reduction in hospital stay compared with initiation between 24 and 48 h [15].

Pain reduction in the immediate postoperative phase also favored earlier initiation. Iwakiri et al. (2020) found significantly lower pain at rest within the first 72 h in patients who began range-of-motion exercises on postoperative day 1 compared with day 7 [26]. Comparable early pain reductions were reported by Labraca et al. (2011) and Gadhavi et al. (2025), both demonstrating significantly lower Visual Analog Scale scores in early groups [28,29].

Improvements in knee range of motion (ROM) during hospitalization or early follow-up were reported by Labraca et al. (2011) and Gadhavi et al. (2025), both demonstrating significantly greater flexion and improved extension in early initiation groups [28,29]. Muscle strength outcomes were also favorable in early groups in both studies, with significantly greater quadriceps and hamstring strength observed during the inpatient recovery phase [28,29]. Additionally, Labraca et al. (2011) reported a higher proportion of patients achieving normal gait and balance at discharge according to the Tinetti test [29].

In contrast, Thwin et al. (2024) found no significant differences in ambulatory distance at discharge between early and later initiation groups [15].

Overall, the evidence consistently supports early rehabilitation for improving acute recovery indicators, particularly hospital length of stay, early pain control, short-term ROM, and early muscle strength.

#### 3.2.2. Medium- and Long-Term Functional Outcomes and Safety

The impact of earlier rehabilitation initiation on medium- and long-term functional outcomes was less consistent across studies.

Iwakiri et al. (2020) reported no significant differences in WOMAC scores at 3 months, 1 year, or 2 years despite early postoperative pain benefits [26]. Similarly, Liebs et al. (2012), in a multicenter randomized trial comparing initiation on postoperative day 6 versus day 14, found no statistically significant differences in WOMAC function, pain, stiffness, quality of life, or patient satisfaction at any follow-up point up to 24 months [27]. Although trends occasionally favored earlier initiation in the TKA subgroup, these differences did not reach statistical significance.

Thwin et al. (2024) also reported no significant differences in patient-reported outcomes, including Oxford Knee Score and Knee Society Score, at 3 months [15]. In contrast, Gadhavi et al. (2025) observed significantly better WOMAC scores at 3 months in the early initiation group; however, this finding has not been replicated in other trials and should therefore be interpreted cautiously [28].

Importantly, none of the included studies reported increased postoperative complications associated with earlier initiation of rehabilitation [15,26,27,28], suggesting that early initiation appears safe within the studied time frames.

Taken together, the evidence indicates that the primary benefits of earlier rehabilitation initiation are concentrated in the immediate postoperative period, whereas sustained long-term functional superiority has not been consistently demonstrated.

## 4. Discussion

This systematic review examined whether earlier initiation of structured postoperative rehabilitation following primary total knee arthroplasty (TKA) results in superior functional outcomes compared with later initiation. The synthesis of the included studies indicates that earlier rehabilitation consistently improves short-term postoperative recovery parameters (particularly hospital length of stay, early pain control, short-term range of motion, and early muscle strength) while evidence for sustained long-term functional superiority remains inconsistent [15,26,27,28,29].

The interpretation of these findings should consider the typical clinical profile of patients undergoing total knee arthroplasty, who are predominantly older adults with advanced knee osteoarthritis, often presenting with comorbidities and reduced baseline function. Postoperatively, these patients commonly experience pain, joint stiffness, and muscle inhibition, which influence the response to rehabilitation. In this context, early-phase interventions primarily target pain control, joint mobility, and ambulation, whereas later stages focus on strength recovery and functional independence.

Reduction in hospital length of stay was one of the most consistent findings across studies. Both randomized trials and the observational cohort reported shorter hospitalization in early initiation groups [15,28,29]. These findings align with broader enhanced recovery after surgery (ERAS) principles, in which early mobilization is considered a central component of accelerated recovery pathways [5,31].

Early postoperative pain control also favored earlier initiation. Iwakiri et al. (2020) reported significantly lower pain at rest within the first 72 h in patients beginning range-of-motion exercises on postoperative day 1 compared with day 7 [26]. Similar reductions in early pain were observed by Labraca et al. (2011) and Gadhavi et al. (2025) [28,29]. These findings are biologically plausible, as early joint mobilization may reduce periarticular stiffness, promote synovial circulation, and facilitate movement-induced analgesia [32].

Short-term improvements in knee range of motion and muscle strength further support the rationale for early intervention. Labraca et al. (2011) and Gadhavi et al. (2025) reported significantly greater flexion, improved extension, and superior quadriceps and hamstring strength in early initiation groups [28,29]. Early activation of the quadriceps may mitigate arthrogenic muscle inhibition, a well-documented phenomenon following TKA characterized by reflexive suppression of muscle activation secondary to joint effusion and inflammation [14,33].

Despite these acute benefits, the evidence does not consistently demonstrate superior long-term functional outcomes. Iwakiri et al. (2020) found no significant differences in WOMAC scores at 3 months, 1 year, or 2 years [26]. Similarly, Liebs et al. (2012) reported no significant differences in WOMAC function, pain, stiffness, or quality-of-life measures up to 24 months when comparing initiation on postoperative day 6 versus day 14 [27]. Thwin et al. (2024) also found no differences in Oxford Knee Score or Knee Society Score at 3 months [15]. Although Gadhavi et al. (2025) reported better WOMAC scores at 3 months in the early group, this finding has not been replicated in other randomized trials and should therefore be interpreted cautiously [28].

This pattern suggests that earlier rehabilitation may accelerate the initial recovery trajectory without substantially modifying the ultimate functional plateau achieved after TKA. Similar convergence of functional outcomes over time has been described in studies comparing rehabilitation intensity and supervision models following TKA [9,34].

Importantly, none of the included studies reported increased postoperative complications associated with earlier rehabilitation initiation [15,26,27,28]. This supports the safety of early mobilization in medically stable patients and is consistent with previous literature demonstrating low complication risk associated with early postoperative mobilization [5].

From a clinical perspective, these findings support early initiation of structured physiotherapy as part of standard postoperative management following primary TKA. Shorter hospital stays and improved early recovery parameters may have implications for healthcare resource utilization and patient throughput. However, clinicians should not assume that earlier initiation alone guarantees superior long-term functional outcomes. Factors such as rehabilitation quality, intensity, adherence, psychosocial variables, and baseline functional status likely exert greater influence on sustained recovery [9]. Hospital length of stay should be interpreted cautiously, as it reflects healthcare system and organizational factors rather than direct functional recovery.

Several limitations must be acknowledged. There was heterogeneity in definitions of “early” and “delayed” initiation across studies, ranging from initiation within 12 h to postoperative day 6, with comparator groups beginning up to postoperative day 14. Outcome measures differed (WOMAC, OKS, KSS), and follow-up durations varied from discharge to 24 months, precluding quantitative pooling and necessitating narrative synthesis. Although most randomized trials demonstrated moderate to high methodological quality according to JBI criteria, allocation concealment and blinding were not consistently reported. The inclusion of one retrospective cohort study introduces potential residual confounding [15]. Important clinical variables such as surgical technique, implant type, perioperative protocols, and patient-specific factors were not consistently reported across studies, limiting clinical applicability. A major limitation of this review is the small number of included studies (*n* = 5). It reflects the limited research specifically isolating timing of rehabilitation initiation as the primary independent variable. This limits the robustness, generalizability, and external validity of the findings. Therefore, conclusions should be interpreted with caution.

Despite these limitations, this review has notable strengths. It addresses a focused and clinically relevant question, applies consistent inclusion criteria centered specifically on timing of rehabilitation initiation, and synthesizes evidence across different healthcare systems. By distinguishing between accelerated early recovery and long-term functional equivalence, the findings clarify an important nuance in postoperative rehabilitation research. An additional limitation is the absence of a universally standardized definition of “early” rehabilitation across studies, which limited direct comparability between intervention timings.

Although subgroup-specific TKA data were extracted from the study by Liebs et al., the inclusion of mixed arthroplasty populations may still limit comparability across studies. The original article did not explicitly report whether the subgroup analysis was pre-planned, which should be considered when interpreting the findings.

In summary, earlier initiation of structured postoperative rehabilitation after primary TKA appears to safely enhance short-term recovery, reducing hospital stay and improving early pain, range of motion, and muscle strength [15,26,28,29]. However, consistent long-term functional superiority has not been demonstrated [15,26,27]. Early rehabilitation should therefore be viewed as a strategy to accelerate recovery rather than to fundamentally alter long-term functional outcomes. From a clinical perspective, these findings support the implementation of early rehabilitation strategies to enhance short-term recovery and reduce hospital length of stay. However, clinicians should recognize that early initiation alone may not determine long-term outcomes, which are likely influenced by rehabilitation quality, patient adherence, and individual clinical characteristics. Future research should focus on standardized definitions of rehabilitation timing, long-term follow-up, and stratification according to patient profiles to identify subgroups that may benefit most from early intervention.

## 5. Conclusions

Early initiation of structured postoperative rehabilitation following primary total knee arthroplasty is associated with improved short-term recovery outcomes, including reduced hospital length of stay, lower early postoperative pain, and improved range of motion and muscle strength during the acute postoperative phase. These findings are consistent across the included studies, despite variability in rehabilitation protocols and timing definitions.

However, no consistent superiority was observed in medium- or long-term patient-reported functional outcomes, suggesting that the benefits of early rehabilitation are primarily limited to the initial stages of recovery. Over time, functional outcomes appear to converge regardless of the timing of rehabilitation initiation.

Taken together, these results indicate that early rehabilitation contributes to accelerating the early recovery trajectory without significantly modifying the overall functional outcome achieved after surgery. These findings provide a clearer understanding of the role of rehabilitation timing in postoperative care following total knee arthroplasty.

## Figures and Tables

**Table 3:** Characteristics of the studies included in the systematic review.

	Typology/Main Objective	Participants	Variables/Instruments	Main Findings	International Banking Institute (JBI)
Iwakiri et al. [26]	Design: Experimental Objective: To examine whether the timing of initiation of postoperative range of motion (ROM) exercises after total knee arthroplasty (TKA)—starting on postoperative day 1 versus day 7—affects postoperative pain, swelling, and ROM recovery, in order to determine the optimal timing for initiating ROM exercises following TKA	*N* = 109 Sex (f/m): 89/20 Age: 75.3 ± 6.8 years	**Postoperative pain at rest:** Visual Analog Scale (VAS, 0–100 mm) **Knee range of motion (flexion/extension):** Clinical angle measurement (goniometry) **Thigh swelling**: Thigh circumference measurement (superior patellar border and 5 cm proximal) with postoperative/preoperative ratio calculation **Knee function and symptoms:** WOMAC (Western Ontario and McMaster Universities Osteoarthritis Index) **Postoperative complications:** Clinical record (surgical site infection, peroneal nerve palsy, deep vein thrombosis, wound complications)	Postoperative pain at rest was significantly lower in the group that started ROM exercises on postoperative day 1 compared with the group that started on day 7, between 18 and 72 h postoperatively (*p* < 0.05). There were no significant differences in knee range of motion (flexion and extension) at any follow-up time point between the two groups (*p* > 0.05). There were no significant differences in thigh swelling between groups at any postoperative time point (*p* > 0.05). There were no significant differences in WOMAC scores at 3 months, 1 year, or 2 years (*p* > 0.05). There were no significant differences in rescue analgesia consumption between groups (*p* > 0.05). No major complications were reported (surgical site infection, peroneal nerve palsy, or deep vein thrombosis) in either group.	9/11
Liebs et al. [27]	Design: Experimental Objective: To evaluate whether early initiation of hydrotherapy (postoperative day 6) compared with late initiation (postoperative day 14) after total knee arthroplasty (TKA) or total hip arthroplasty (THA) improves postoperative physical function, primarily measured by the WOMAC function subscale, and to assess its effects on pain, stiffness, quality of life, and patient satisfaction.	*N* = 465 Only the TKA subgroup was considered for analysis (*n* = 185). Sex (f/m): 309/156 Age: ≈68.9 years	**Physical function:** WOMAC function subscale (VAS; 0–100, lower = better) liebs2012 **Pain:** WOMAC pain subscale liebs2012 **Stiffness:** WOMAC stiffness subscale liebs2012 **Quality of life (physical component):** SF-36 Physical Component Summary liebs2012 **Joint-specific severity/function:** Lequesne Hip/Knee Score liebs2012 **Patient satisfaction:** satisfaction question (% “very satisfied”)	No statistically significant differences were found between early (day 6) and late (day 14) hydrotherapy initiation in physical function measured by the WOMAC function subscale at any follow-up point (3, 6, 12, and 24 months; *p* > 0.05). In the total knee arthroplasty (TKA) subgroup, patients who started hydrotherapy early showed better mean functional scores, but these differences were not statistically significant at any time point (*p* > 0.05). In the total hip arthroplasty (THA) subgroup, slightly better results were observed in the late-start group, but again no statistically significant differences were found (*p* > 0.05). No significant differences were observed between groups in pain, stiffness, quality of life, Lequesne score, or patient satisfaction at any follow-up (*p* > 0.05). Overall, early hydrotherapy did not demonstrate statistically significant advantages over late initiation.	9/11
Thwin et al. [15]	Design: Retrospective cohort study Objective: To investigate whether ultra-early physical therapy (<12 h postoperatively) after total knee arthroplasty (TKA) improves functional outcomes and reduces length of stay compared with early (<24 h) or later (24–48 h) initiation.	*N* = 569 Sex (f/m): 361/208 Age: 67.7± 7.8	**Length of stay (LOS):** Days from admission to discharge **Passive range of motion (PROM):** Clinical measurement in degrees (discharge and 3 months) **Ambulatory distance at discharge:** Distance in meters **Patient-reported function:** Oxford Knee Score (OKS) **Clinical function:** Knee Society Score (KSS) **Functional component:** KSS Function score	Patients who initiated physical therapy within 24 h had a significantly shorter hospital stay than those who started between 24 and 48 h (4.87 vs. 5.34 days; *p* = 0.002). In subgroup analysis, patients who started physical therapy within 12 h had a slightly shorter length of stay compared to those who started between 12 and 24 h (4.75 vs. 4.96 days; *p* = 0.009), although the difference was small and likely not clinically significant. No significant differences were observed between groups in range of motion at discharge or at 3 months (*p* > 0.05). No significant differences were found in ambulatory distance at discharge (*p* = 0.626). No significant differences were observed in Oxford Knee Score or Knee Society Score at 3 months (*p* > 0.05). There were no differences in complication rates between groups.	
Gadhavi et al. [28]	Design: Prospective observational study Objective: To compare the effects of initiating physiotherapy within 24 h versus after 48 h following primary total knee arthroplasty, assessing pain, range of motion, function, and length of hospital stay.	*N* = 100 Sex (f/m): 62/38 Age: 62.4 ± 7.2 years	**Pain**: Visual Analog Scale (VAS 0–10) **Range of motion**: Goniometric measurement **Function**: WOMAC (0–96; lower score = better function) **Length of stay**: Days from surgery to discharge **Complications**: Clinical record	Patients who initiated physiotherapy within 24 h reported lower pain on day 3 (3.2 vs. 5.1; *p* < 0.001). They achieved greater range of motion at week 2 (85° vs. 72°; *p* < 0.01). They demonstrated better functional outcomes at 3 months according to WOMAC (28.5 vs. 35.7; *p* = 0.002). Length of stay was significantly shorter in the early group (4.8 vs. 6.3 days; *p* < 0.001). No significant differences in complication rates were observed.	8/11
Labarca et al. [29]	Design: Experimental Objective: To compare the benefits of initiating rehabilitation within 24 h versus 48–72 h after primary total knee arthroplasty for osteoarthritis.	*N* = 306 Sex (f/m): 211/62 Age: 66.02 ± 5.37 years	**Pain:** Visual Analog Scale (VAS 0–10) **Range of motion**: Goniometric measurement **Muscle strength**: Daniels & Worthingham manual muscle test (0–5) **Functional autonomy:** Barthel Index **Gait and balance:** Tinetti test **Hospital length of stay:** Days to discharge **Number of rehabilitation sessions until discharge**	The group that initiated rehabilitation within 24 h had a significantly shorter hospital stay (6.37 vs. 8.46 days; *p* < 0.001). They required fewer rehabilitation sessions until discharge (14.92 vs. 19.87; *p* < 0.001). They reported lower postoperative pain (VAS 3.01 vs. 5.36; *p* < 0.027). They achieved greater knee flexion (88.11° vs. 71.82°; *p* < 0.012) and better extension (0.68° vs. 2.80°; *p* < 0.035). Muscle strength was significantly higher in the early group for both quadriceps (3.91 vs. 3.01; *p* < 0.042) and hamstrings (4.02 vs. 2.97; *p* < 0.041). More patients in the early group achieved normal gait and balance according to the Tinetti test (*p* < 0.05). No significant differences were found in Barthel Index scores.	

## Data Availability

No new data were created or analyzed in this study. Data sharing is not applicable to this article.

## Abbreviations

The following abbreviations are used in this manuscript:

TKA	Total Knee Arthroplasty
THA	Total Hip Arthroplasty
ROM	Range of Motion
LOS	Length of Stay
VAS	Visual Analog Scale
WOMAC	Western Ontario and McMaster Universities Osteoarthritis Index
OKS	Oxford Knee Score
KSS	Knee Society Score
SF-36	Short Form Health Survey (36-item)
PROMs	Patient-Reported Outcome Measures
JBI	Joanna Briggs Institute
PRISMA	Preferred Reporting Items for Systematic Reviews and Meta-Analyses
ERAS	Enhanced Recovery After Surgery
SD	Standard Deviation
TUG	Timed Up and Go Test
